# Regular Medicine and Homœopathy—Remark

**Published:** 1871-09

**Authors:** 


### Regular Medicine and Homœopathy.

We have received numerous communications on the above subject, all agreeing that it is quite unnecessary, and even absurd to answer or explain to medical men, the reason why physicians do not, and cannot associate with the various

"ists" and "ics," or to expose to further ridicule the legal errors in the communication on the above subject, or in any way make serious lengthened reply to the paper. If our readers and correspondents will forgive us for publishing the paper for professional reading, and for suppressing their very complete and satisfactory rejoinders, we will regard the unpardonable sins of editoral life blotted out, and solemnly promise with penitence and prayer, to never again thus trespass upon their indulgence.

